# Neuroimaging findings and neurological manifestations in hospitalized COVID-19 patients: Impact of cancer and ventilatory support status

**DOI:** 10.1371/journal.pone.0283614

**Published:** 2023-03-24

**Authors:** Lily McCarthy, Oleksandr Khegai, Jonathan Goldstein, Puneet Belani, Puneet Pawha, Shingo Kihira, Brian Mathew, Kapil Gururangan, Qing Hao, Anuradha Singh, Allison Navis, Bradley N. Delman, Nathalie Jette, Priti Balchandani

**Affiliations:** 1 Icahn School of Medicine at Mount Sinai, New York, NY, United States of America; 2 BioMedical Engineering and Imaging Institute, Icahn School of Medicine at Mount Sinai, New York, NY, United States of America; 3 Department of Neurology, Icahn School of Medicine at Mount Sinai, New York, NY, United States of America; 4 Department of Diagnostic, Molecular and Interventional Radiology, Icahn School of Medicine at Mount Sinai, New York, NY, United States of America; 5 Department of Population Health Science and Policy, Icahn School of Medicine at Mount Sinai, New York, NY, United States of America; Acknowledgments; Foshan Sanshui District People’s Hospital, CHINA

## Abstract

**Introduction:**

Coronavirus 2019 (COVID-19) is known to affect the central nervous system. Neurologic morbidity associated with COVID-19 is commonly attributed to sequelae of some combination of thrombotic and inflammatory processes. The aim of this retrospective observational study was to evaluate neuroimaging findings in hospitalized COVID-19 patients with neurological manifestations in cancer versus non-cancer patients, and in patients with versus without ventilatory support (with ventilatory support defined as including patients with intubation and noninvasive ventilation). Cancer patients are frequently in an immunocompromised or prothrombotic state with side effects from chemotherapy and radiation that may cause neurological issues and increase vulnerability to systemic illness. We wanted to determine whether neurological and/or neuroimaging findings differed between patients with and without cancer.

**Methods:**

Eighty adults (44 male, 36 female, 64.5 ±14 years) hospitalized in the Mount Sinai Health System in New York City between March 2020 and April 2021 with reverse-transcriptase polymerase chain reaction-confirmed COVID-19 underwent magnetic resonance imaging (MRI) during their admissions. The cohort consisted of four equal subgroups based on cancer and ventilatory support status. Clinical and imaging data were acquired and analyzed.

**Results:**

Neuroimaging findings included non-ischemic parenchymal T2/FLAIR signal hyperintensities (36.3%), acute/subacute infarcts (26.3%), chronic infarcts (25.0%), microhemorrhages (23.8%), chronic macrohemorrhages (10.0%), acute macrohemorrhages (7.5%), and encephalitis-like findings (7.5%). There were no significant differences in neuroimaging findings between cancer and non-cancer subgroups. Clinical neurological manifestations varied. The most common was encephalopathy (77.5%), followed by impaired responsiveness/coma (38.8%) and stroke (26.3%). There were significant differences between patients with versus without ventilatory support. Encephalopathy and impaired responsiveness/coma were more prevalent in patients with ventilatory support (p = 0.02). Focal weakness was more frequently seen in patients without ventilatory support (p = 0.01).

**Discussion:**

This study suggests COVID-19 is associated with neurological manifestations that may be visible with brain imaging techniques such as MRI. In our COVID-19 cohort, there was no association between cancer status and neuroimaging findings. Future studies might include more prospectively enrolled systematically characterized patients, allowing for more rigorous statistical analysis.

## Introduction

The novel coronavirus severe acute respiratory syndrome 2 (SARS-CoV-2), causative agent for coronavirus 2019 disease (COVID-19), was initially considered a respiratory disease [1]. With broader clinical experience it has been found to affect multiple organ systems including the central and peripheral nervous systems, leading to diverse neurological manifestations [2]. Among the most common neurological symptoms are anosmia, dysgeusia, myalgia, headache, dizziness, and syncope [3]. Altered mental status, confusion, and delirium are frequently seen in hospitalized patients [4]. Meningoencephalitis and acute hemorrhagic encephalopathy, while rare, have also been documented in COVID-19 [4, 5]. Stroke is also prevalent, including acute and subacute infarcts, cerebral microhemorrhages, and spontaneous intracranial hemorrhage [6]. The diagnosis of many of these neurological manifestations may be confirmed with neuroimaging modalities including magnetic resonance imaging (MRI), which has revealed acute and hemorrhagic stroke, leptomeningeal enhancement, encephalomyelitis-like pattern, and white matter hyperintensities in COVID-19 cohorts [7].

Despite the growing number of studies on the implications of COVID-19 on the central nervous system (CNS), understanding of this disease and its effects on neurological functioning in different patient populations is still evolving [8–10]. To date, few studies have explored neurological and neuroimaging abnormalities in specific patient populations, including cancer patients who have a higher baseline risk of prothrombotic events, infection, and other complications. Thus, the differential neurological effects of COVID-19 on patients with various diseases such as cancer are still relatively unknown. The purpose of this retrospective observational study was to determine whether a pre-existing diagnosis of non-CNS primary cancer affected neuroimaging findings associated with COVID-19, with a cohort composed of four subgroups: patients with ventilatory support and with cancer, without ventilatory support and with cancer, with ventilatory support and without cancer, and without ventilatory support and without cancer. Ventilatory support was defined as encompassing patients with intubation and with noninvasive ventilation. All patients in our cohort were hospitalized with COVID-19, and all had neurological manifestations. Apart from these two main similarities between patients, our cohort was largely heterogeneous. Patients in the cancer subgroups had a range of non-CNS cancers, as we endeavored to study the potential effects of many different types of cancer as opposed to one specific category. The rationale of this study was to report the neurological manifestations and neuroimaging findings in each of these four subgroups, providing a foundation for future investigation into the comorbid ramifications of COVID-19 on the brain, particularly in the context of non-CNS neoplasms.

## Methods

Institutional Review Board (IRB) approval from the Program for Protection of Human Subjects at the Icahn School of Medicine at Mount Sinai was obtained for this retrospective observational study (GCO 15–0219). The requirement for informed consent was waived by the ethics committee for the purposes of retrospective analysis. As we were building a tool and performing retrospective analyses of medical data, the research necessitated use of PHI for the purpose of building predictive models and classifiers. The data was explored in terms of the extracted features to create visualizations that summarize large patient cohorts and predictive models to better risk-stratify patient populations. The waiver or alteration did not adversely affect the rights and welfare of the participants since no data was disclosed to another institution without permission and all patient identifiers were maintained in a secure digital environment.

Patient population–Eighty adults hospitalized with COVID-19 between March 4, 2020 and April 8, 2021 who underwent brain MRI at the Mount Sinai Health System in New York and experienced neurological manifestations were randomly identified. The cohort consisted of four subgroups, with 20 patients in each: cancer patients with ventilatory support, cancer patients without ventilatory support, non-cancer patients with ventilatory support, and non-cancer patients without ventilatory support. A diagnosis of COVID-19 was confirmed in all patients by reverse-transcriptase polymerase chain reaction (PCR) assay.

Imaging methods–As a multihospital review, exact sequences could vary. In general patients underwent scanning at 1.5T including axial T2, axial T2 FLAIR, axial GRE, sagittal T1, and diffusion weighted imaging (DWI) with apparent diffusion coefficient (ADC) maps. Three fellowship-trained neuroradiologists with 5, 15, and 22 years of experience and a senior (fourth-year) radiology resident reviewed the MRI scans using a randomized blinded multiple paired readers approach. Readers were blinded to cancer diagnosis and ventilatory support status. Brain images were scrutinized for abnormalities including non-ischemic parenchymal T2/FLAIR hyperintensities, acute/subacute and chronic infarcts, and macro- and microhemorrhages on the basis of visual qualitative assessment. Diffusion abnormalities were investigated on the basis of ADC maps. Any inter-reader discrepancies were subsequently adjudicated through consensus among the three fellowship-trained neuroradiologists.

Data extraction–Age, sex, cancer diagnosis, and ventilatory support status were extracted from the Mount Sinai Clinical Intelligence Center database. The Mount Sinai Health System Epic electronic medical record was used to gather the neurological and non-neurological clinical characteristics of the COVID-19 patients. All hospital records (admission and discharge summaries, progress notes, consult notes, nursing notes, allied health professional notes) were reviewed for the presence of neurological symptoms (e.g., anosmia), signs (e.g., coma) or diagnoses (e.g., stroke). Patients noted to have delirium, confusion or encephalopathy were all categorized under the “encephalopathy” category. Anxiety, depression and psychosis-related manifestations were also extracted. Both clinical history and physical findings were reviewed to ascertain neurological manifestations. Chart abstraction was initially done by two chart reviewers trained by a board-certified neurologist, and all diagnoses were confirmed in a final review by the neurologist.

Data analysis–A Fisher’s exact test was performed to determine whether there were statistically significant differences between cancer and non-cancer subgroups. The Fisher’s exact test is a statistical significance test used in the analysis of contingency tables and is often used when sample sizes are small, as was the case for cohort size of the current study. Detailed analysis spreadsheets are included as supplementary materials.

## Results

Eighty patients (44 male, 36 female, 64.5 ±14 years) were included. The cohort was composed of four subgroups: 20 cancer patients with ventilatory support (12 male, 8 female, 65.8 ±13.3 years), 20 cancer patients without ventilatory support (11 male, 9 female, 64.4 ±16.6 years), 20 non-cancer patients with ventilatory support (12 male, 8 female, 61.8 ±12.8 years), and 20 non-cancer patients without ventilatory support (9 male, 11 female, 65.9 ±12.7 years). Out of the 20 cancer patients with ventilatory support, 12/20 were intubated and 8/20 were on high level oxygen supply (non-invasive ventilation, including bilevel positive airway pressure).

### Neurological manifestations

Neurological manifestations were diverse (Table 1). The three most common were encephalopathy (77.5%), followed by impaired responsiveness/coma (38.8%) and ischemic stroke (26.3%). Other neurological and psychiatric manifestations included headache, seizures/status epilepticus, hemorrhagic stroke, hyposmia/anosmia, visual symptoms, hypogeusia/ageusia, dysphagia, dysarthria, focal weakness, abnormal movements or tone, parkinsonism, dysmetria/incoordination, gait abnormality/ataxia, critical illness neuropathy/myopathy, acute inflammatory demyelinating polyneuropathy/Guillain-Barré syndrome, numbness (extremities), depression, anxiety and psychosis. There were no statistically significant differences between any neurological manifestations in cancer versus non-cancer patients. There were several significant differences between patients with versus without ventilatory support. Encephalopathy was more common in patients with ventilatory support (p = 0.02), as was impaired responsiveness/coma (p<0.01). Focal weakness was more common in patients without ventilatory support (p = 0.01).

**Table 1:** Neurological manifestation listed by highest to lowest frequency.

	All (N = 80) N (%)	Cancer N = 40	Non-cancer N = 40	Ventilatory support N = 40	No ventilatory support N = 40	Non-cancer, no ventilatory support N = 20	Non-cancer, ventilatory support N = 20	Cancer, no ventilatory support N = 20	Cancer, ventilatory support N = 20
**Encephalopathy**	62 (77.5)	27	35	36	26	16	19	10	17
**Impaired responsiveness/coma**	31 (38.8)	12	19	26	5	4	15	1	11
**Stroke (ischemic)**	21 (26.3)	9	12	10	11	7	5	4	5
**Headache**	17 (21.3)	9	8	5	12	7	1	5	4
**Weakness (focal)**	16 (20.0)	8	8	3	13	5	3	8	0
**Seizures/status epilepticus**	13 (16.3)	6	7	8	5	2	5	3	3
**Critical illness neuropathy/myopathy**	11 (13.8)	4	7	8	3	1	6	2	2
**Abnormal movements or tone**	10 (12.5)	6	4	5	5	3	1	2	4
**Visual symptoms**	10 (12.5)	4	6	2	8	5	1	3	1
**Dysarthria**	9 (11.3)	5	4	4	5	2	2	3	2
**Numbness (extremities)**	8 (10.0)	5	3	4	4	2	1	2	3
**Anxiety**	7 (8.8)	4	3	2	5	2	1	3	1
**Depression**	7 (8.8)	4	3	4	3	2	1	1	3
**Gait abnormality/ataxia**	6 (7.5)	5	1	1	5	1	0	4	1
**Psychosis/hallucination**	5 (6.3)	3	2	3	2	0	2	2	1
**Stroke (hemorrhagic)**	3 (3.8)	3	0	2	1	0	0	1	2
**Dysmetria/incoordination**	3 (3.8)	1	2	1	2	1	1	1	0
**Hyposmia/anosmia**	2 (2.5)	2	0	0	2	0	0	2	0
**Hypogeusia/ageusia**	2 (2.5)	1	1	0	2	1	0	1	0
**Dysphagia**	2 (2.5)	2	0	1	1	0	0	1	1
**Parkinsonism**	1 (1.3)	0	1	0	1	1	0	0	0
**Acute inflammatory demyelinating polyneuropathy/Guillain-Barré syndrome**	1 (1.3)	1	0	1	0	0	0	0	1

Neurological manifestations in all cancer patients were compared to those in all non-cancer patients, and all patients with ventilatory support were compared to patients without ventilatory support. There were no statistically significant differences between neurological manifestations in cancer versus non-cancer patients. However, there were statistically significant differences between patients with versus without ventilatory support. Encephalopathy and impaired responsiveness/coma were more common in patients with ventilatory support, while focal weakness was more common in patients without ventilatory support.

### Neuroimaging findings

MRI exams were performed on average 13.2 days (standard deviation ±14.1 days) after the diagnosis of COVID-19. The delay between the diagnosis of COVID-19 and MRI exam was often due to the patients’ health conditions and clinical necessity, including the need to stabilize patients. The spectrum of neuroimaging findings was heterogeneous (Table 2). Of the 80 patients imaged, 36.3% of patients had non-ischemic parenchymal T2/FLAIR signal hyperintensities, 26.3% had acute/subacute infarcts, 25.0% had chronic infarcts, 23.8% had microhemorrhages, 10.0% had chronic macrohemorrhages, 7.5% had acute macrohemorrhages, and 7.5% had encephalitis-like findings. There were no clinically significant qualitative neuroimaging findings in 32.5% of patients, including 6 cancer patients with ventilatory support, 8 cancer patients without ventilatory support, 5 non-cancer patients with ventilatory support, and 7 non-cancer patients without ventilatory support. There were no statistically significant differences between any of these neuroimaging findings in cancer and non-cancer groups or between groups with and without ventilatory support. Additional information about imaging findings in each of the four subgroups can be found below.

**Table 2:** Neuroimaging findings.

	All (N = 80) N (%)	Cancer N = 40	Non-cancer N = 40	Ventilatory support N = 40	No ventilatory support N = 40	Non-cancer, no ventilatory support N = 20	Non-cancer, ventilatorys upport N = 20	Cancer, no ventilatory support N = 20	Cancer, ventilatory support N = 20
**T2 hyperintensity**	29 (36.3)	15	14	18	11	4	10	7	8
**Acute/subacute infarct**	21 (26.3)	10	11	8	13	9	2	4	6
**Chronic infarct**	20 (25.0)	9	11	13	7	3	8	4	5
**Acute macrohemorrhage**	6 (7.5)	2	4	4	2	1	3	1	1
**Chronic macrohemorrhage**	8 (10.0)	7	1	3	5	1	0	4	3
**Microhemorrhage**	19 (23.8)	7	12	11	8	5	7	3	4
**Encephalitis-like findings**	6 (7.5)	1	5	5	1	1	4	0	1
**No findings**	26 (32.5)	14	12	11	15	7	5	8	6

The most common neuroimaging findings are listed above for each of the four subgroups, with hyperintensities and infarcts (acute/subacute and chronic) emerging as the most prevalent. No significant differences between neuroimaging findings in cancer versus non-cancer subgroups were observed.

#### Non-ischemic parenchymal T2/FLAIR signal hyperintensities

Non-ischemic parenchymal T2/FLAIR signal hyperintensities were the most common neuroimaging finding in our cohort. The prevalence of hyperintensities was not significantly different between cancer versus non-cancer patients (15/40 cancer patients versus 14/40 non-cancer patients). Hyperintensities were slightly higher in patients with ventilatory support than in those without ventilatory support (18/40 patients with ventilatory support versus 11/40 patients without ventilatory support), but this difference was not statistically significant. When stratified by subgroup, there were 8 patients with hyperintensities in the cancer subgroup with ventilatory support, 7 in the cancer subgroup without ventilatory support, 10 in the non-cancer subgroup with ventilatory support, and 4 in the non-cancer subgroup without ventilatory support.

#### Acute/Subacute infarcts and chronic infarcts

**Fig 1:**
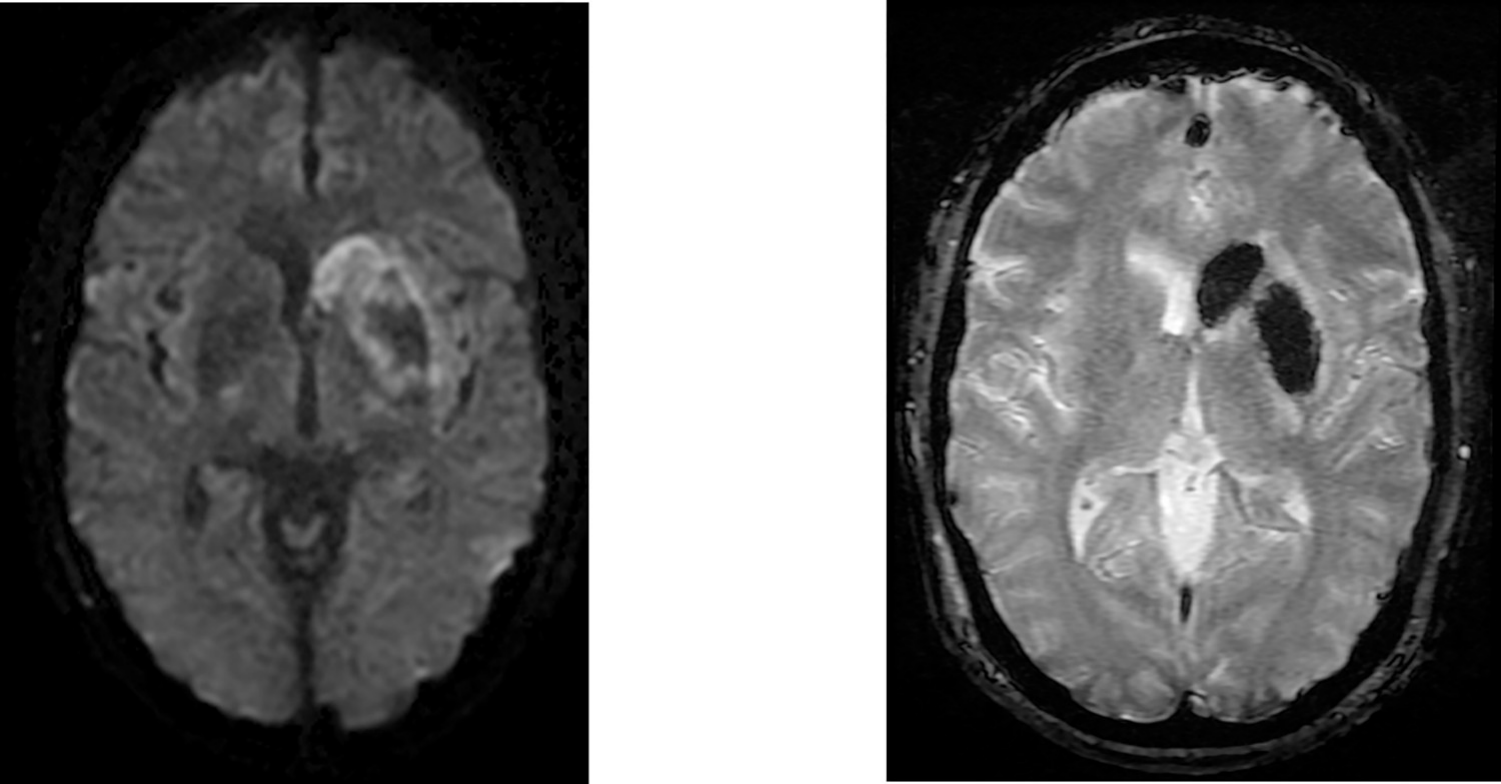
Imaging findings in a 54 y.o. female COVID-19 patient with an endometrial carcinoma who was not receiving ventilatory support. An acute infarct in the left basal ganglia is shown in the DWI image on the left. The heterogeneous signal pattern through the infarct bed is explained in part by the axial gradient echo image on the right, which shows susceptibility through much of the same territory implicating hemorrhage conversion.

Acute/subacute and chronic infarcts were also common in our cohort and did not differ between cancer and non-cancer patients (acute/subacute: 10/40 cancer patients versus 11/40 non-cancer patients; chronic: 9/40 cancer patients versus 11/40 non-cancer patients) or between patients with versus without ventilatory support (acute/subacute: 8/40 patients with ventilatory support versus 13/40 patients without ventilatory support; chronic: 13/40 patients with ventilatory support versus 7/40 patients without ventilatory support). There were 6 patients with acute/subacute infarcts in the cancer subgroup with ventilatory support, 4 in the cancer subgroup without ventilatory support, 2 in the non-cancer subgroup with ventilatory support, and 9 in the non-cancer subgroup without ventilatory support. Fig 1 shows a representative acute infarct patient in a patient from the cancer subgroup without ventilatory support. Chronic infarcts were found in 5 cancer patients with ventilatory support, 4 cancer patients without ventilatory support, 8 non-cancer patients with ventilatory support, and 3 non-cancer patients without ventilatory support.

#### Intracranial hemorrhages: Microhemorrhages, chronic macrohemorrhages, and acute macrohemorrhages

Various intracranial hemorrhages were observed, with no meaningful differences between cancer and non-cancer patients (microhemorrhages: 7/40 cancer patients versus 12/40 non-cancer patients; chronic macrohemorrhages: 7/40 cancer patients versus 1/40 non-cancer patients; acute macrohemorrhages: 2/40 cancer patients versus 4/40 non-cancer patients) or between patients with versus without ventilatory support (microhemorrhages: 11/40 patients with ventilatory support versus 8/40 patients without ventilatory support; chronic macrohemorrhages: 3/40 patients with ventilatory support versus 5/40 patients without ventilatory support; acute macrohemorrhages: 4/40 patients with ventilatory support versus 2/40 patients without ventilatory support). Microhemorrhages were identified in 4 cancer patients with ventilatory support, 3 cancer patients without ventilatory support, 7 non-cancer patients with ventilatory support, and 5 non-cancer patients without ventilatory support. Chronic macrohemorrhages were detected in 3 cancer patients with ventilatory support, 4 cancer patients without ventilatory support, and 1 non-cancer patient without ventilatory support, with none in the non-cancer subgroup with ventilatory support. Acute macrohemorrhages were found in 1 cancer patient with ventilatory support, 1 cancer patient without ventilatory support, 3 non-cancer patients with ventilatory support and 1 non-cancer patient without ventilatory support.

#### Encephalitis-like findings

**Fig 2:**
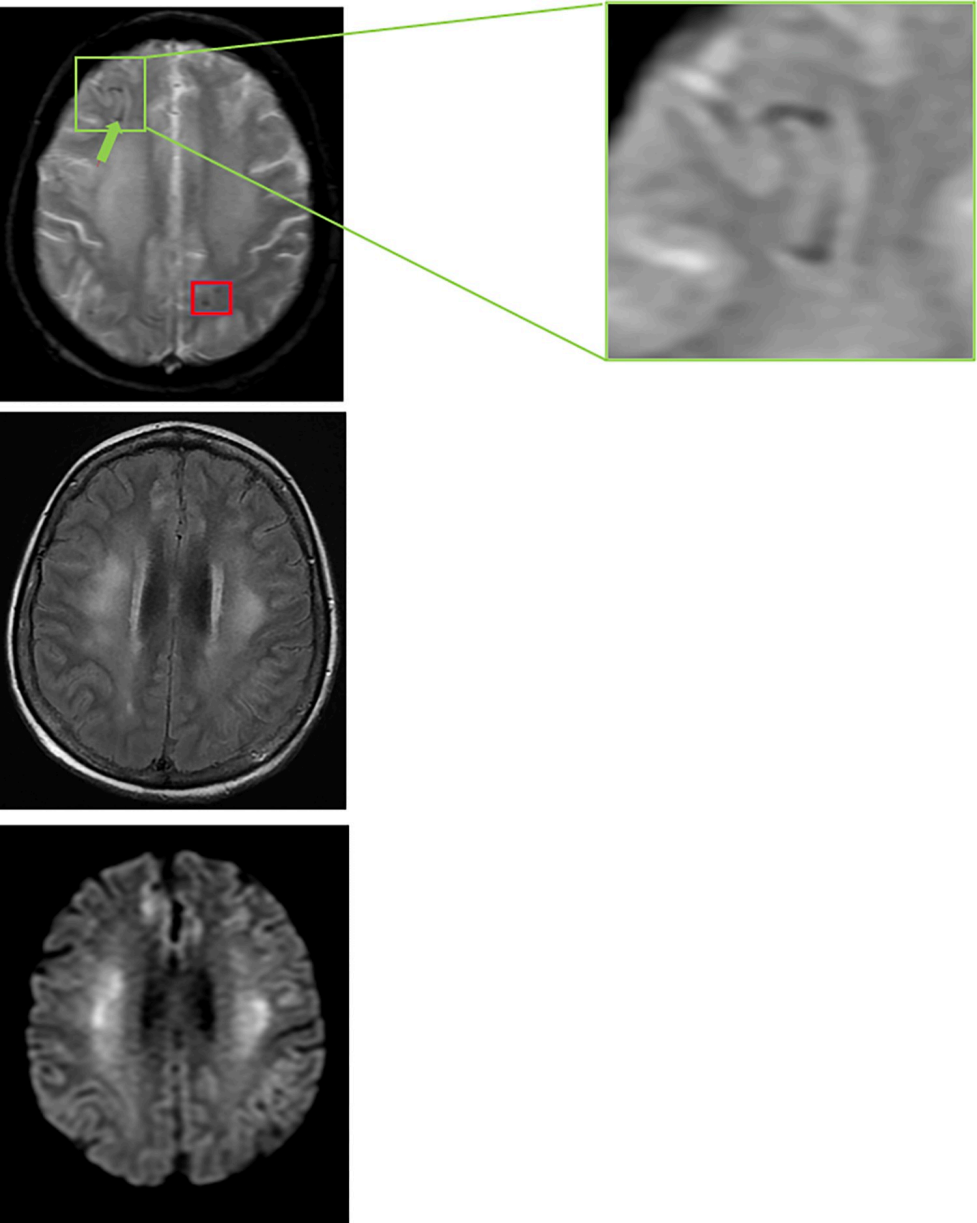
Brain MR images in a 61 y.o. female COVID-19 patient from the non-cancer subgroup who was receiving ventilatory support. Axial gradient echo image shows right frontal subarachnoid hemorrhage (green arrow with enlarged inset showing curvilinear dark signal) and stippled/punctate foci of low signal reflecting left parietal parenchymal microhemorrhage (red box). Bilateral symmetric hyperintensities in the cerebral white matter consistent with encephalitis-like pattern are shown in the axial FLAIR (middle) and DWI (bottom) images.

Encephalitis-like findings was the designation used to reflect imaging appearance of encephalitis of either infectious or inflammatory etiologies. Six patients had encephalitis-like findings on imaging, including 1 cancer patient with ventilatory support, 4 non-cancer patients with ventilatory support, and 1 non-cancer patient without ventilatory support. One case of encephalitis-like finding in a non-cancer patient with ventilatory support is shown in Fig 2. Encephalitis-like findings were less common in patients without ventilatory support, with no cases in the cancer subgroup without ventilatory support and only 1 case in the non-cancer group without ventilatory support. All 6 cases of encephalitis-like findings were imaged at least 25 days after COVID-19 diagnosis.

## Discussion

In this retrospective study, we present data from 80 hospitalized patients with neurological manifestations and neuroimaging findings that arose on presentation or soon after COVID-19 diagnosis. The most pervasive neurological manifestation, was encephalopathy, found in 77.5% of patients, while the leading neuroimaging finding noted in 36.3% was non-ischemic parenchymal T2/FLAIR signal hyperintensities, closely followed by acute/subacute infarction. There were no statistically significant differences between neurological manifestations or neuroimaging findings in cancer versus non-cancer patients.

To our knowledge, this is the first study to directly compare neurologic manifestations and neuroimaging findings in cancer versus non-cancer patients. Previous studies have identified associations between cancers such as primary CNS lymphoma and more severe COVID-19 illness [11]. Patients with cancer, along with cardiovascular diseases, type-2 diabetes, and chronic obstructive pulmonary disease, are also more frequently hospitalized with COVID-19 than the general population [12]. In fact, a study involving data from 322,817 COVID-19 patients (2.3% of whom also had cancer) showed that COVID-19 patients with cancer had a higher disease severity and higher risk of mortality than those without cancer [13]. Specifically, COVID-19 patients with cancer were more likely to have a longer hospital stays, be admitted to the intensive care unit, and receive more mechanical ventilation [13]. The only prior study investigating the relationship between cancer and moderate to severe neurological symptoms in COVID-19 documented that leptomeningeal inflammatory cytokines preceded neurocognitive dysfunction [14]. However, unlike our current investigation, that study did not compare cancer patients to non-cancer patients. Although the limited sample size of our own cohort limits the conclusions that can be drawn about the relationship between cancer and neurological sequelae of COVID-19, we present preliminary findings to clarify the impact of the virus on cancer patients. At least within our small cohort, an underlying diagnosis of cancer did not seem to substantially influence neurological manifestations or neuroimaging findings associated with COVID-19.

The underlying neurological manifestations in our cohort are concordant with those reported in prior studies. For example, an early case series involving three special care centers in China reported dizziness (16.8%), headache (13.1%), dysgeusia (5.6%), and anosmia (5.1%) as the most common symptoms in their cohort of 214 patients, with others including cognitive impairment, ataxia, and stroke [15]. Another Italian multicenter study identified altered mental status (59%) and stroke (31%) as the most frequent neurological symptoms in their cohort of 108 hospitalized patients, similar to our findings [16]. A prospective study of neurological manifestations in 606 hospitalized patients with COVID-19 found that 6.8% had encephalopathy, consistent with our results [17]. In a recent meta-analysis including 19 studies (with 11,324 total patients), fatigue (37%), brain fog (32%) memory issues (27%), attention disorder (22%), myalgia (18%), anosmia (12%), dysgeusia (11%), and headache (10%) were the most prevalent post-COVID-19 neurological symptoms [18]. Hospital admission was correlated with more frequent memory issues, and intensive care unit admission during acute COVID-19 was associated with more prevalent fatigue, anxiety, depression, and sleep disturbances [18]. Another recent meta-analysis found numerous neurological manifestations associated with both the CNS and PNS, including headache, cerebrovascular diseases, altered mental status, encephalopathy, encephalitis, movement disorders, and Guillain-Barre syndrome [19]. More broadly, the impact of COVID-19 on stroke admissions and outcomes is also garnering increasing attention [20–22], with one recent study identifying higher cerebral venous thrombosis mortality in COVID-19 positive patients compared to COVID-19 negative patients [23].

In terms of radiological observations, non-ischemic parenchymal T2/FLAIR signal hyperintensities were most frequent feature in our patients. Hyperintensities were slightly more common in patients with ventilatory support than in those without ventilatory support; however, this difference was not statistically significant. Such non-specific hyperintensities in diverse regions have been widely reported among critically ill COVID-19 patients [24–26]. This neuroimaging abnormality has been linked to hypoxia in some cases [27]. Periods of hypoxia prior to intubation of COVID-19 patients may be similar to what is observed in individuals with high altitude sickness, which also may present with T2/FLAIR signal hyperintensities [28]. These findings persist even given the prevalence of white matter hyperintensities is also known to increase with age [29], which may account for some of the hyperintensities seen in our cohort. Future work will elucidate what percentage of these hyperintensities is due to the normal aging process, COVID-19, or other clinical factors. Infarcts, both acute/subacute (26.3%) and chronic (25%), were also common, seen in a 36/80 patients (45%). A substantial number of COVID-19 patients (40.9%) in a recent neuroimaging study had acute or chronic infarcts, hemorrhages, or additional chronic findings [30]. Acute infarcts were similarly detected in 7/24 (29%) patients with acute neuroimaging findings in another study [31]. Acute infarcts with large clot burden, including large vessel, small vessel, and watershed infarcts, were also routine findings in a recent review of COVID-19 neuroimaging manifestations [8]. Acute infarcts (31%), involving large, small, cardioembolic, and hypoxic-ischemic encephalopathy-related infarcts, were likewise major neuroimaging hallmarks in the multicenter study in Italy [16]. Acute infarcts also topped the list of neuroimaging characteristics of COVID-19 patients in a recent global multicenter study (28%) [32].

Additionally, microhemorrhages (23.8%) were visualized in a sizeable proportion of our cohort, with acute macrohemorrhages (7.5%) and chronic macrohemorrhages (10.0%) seen less frequently. There is increasing literature on of microhemorrhages in COVID-19 patients [33–35]. In a recent small US study, punctuate microhemorrhages were detected in the juxtacortical white matter and corpus callosum in 7/27 patients (26%) [36]. Macrohemorrhages have also been associated with COVID-19 in recent studies [37, 38]. The relatively few numbers of patients with acute and chronic macrohemorrhages in our own cohort made it challenging to infer much about potential variations between different subgroups.

Finally, encephalitis-like findings were recorded in 6/80 patients in our study (7.5%). All but one of these patients were receiving ventilatory support (5/6). A recent study similarly found that COVID-19 patients in their cohort (4.4%) displayed abnormalities associated with encephalopathy on MRI [39]. The fact that most of our own patients with encephalitis-like findings on imaging were more critically ill, therefore requiring ventilatory support, makes sense given that encephalopathy is typically seen in sicker patients and is correlated with poorer outcomes [40]. Encephalopathy is being reported with increasing frequency as a potential consequence of COVID-19 [5]. The relationship between frequency of encephalopathy and severity of disease is supported by a recent review showing that encephalopathy was more common in COVID-19 patients who had more breathing difficulties and required respiratory assistance such as mechanical ventilation [41]. All of our patients with encephalitis-like findings underwent their MRI scans 25 or more days after COVID-19 diagnosis, an extended window that provided time for the wide-ranging brain damage to develop. Further work should corroborate whether this interlude between diagnosis and imaging accounts for the phenomenon observed.

Limitations of our study include the small sample size, a shortcoming to address in future studies with more patients and rigorous statistical analysis. To date, very little has been published on the influence of cancer on neurological abnormalities in individuals with concurrent COVID-19 diagnoses. More literature on cancer patients with COVID-19-related neurological issues will be welcome, given greater susceptibility to infection and mortality among cancer patients [42]. Thus, determining how this comorbidity is impacted and whether there is a relationship between cancer and neurological manifestations seen in the context of COVID-19 would be highly valuable [43]. Another limitation is the delay between COVID-19 diagnosis and MRI due to reasons such as disease severity, clinical necessity, MRI availability, feasibility of scanning acute patients, and other factors. Patients in the intensive care unit with severe COVID-19 requiring ventilatory support were often scanned after a delay to allow for clinical stabilization. An additional limitation relates to neurological manifestations reported in this study. We found that encephalopathy was more prevalent in patients with ventilatory support. However, these patients may have been given ventilatory support in part because they were encephalopathic. We cannot rule out this possible bias. Finally, since this study was retrospective in nature, it did not include controls, so we cannot rule out the possibility that observed abnormalities were due to COVID-19 and not to other etiologies. Nevertheless, the fact that we investigated the relationship between neurological manifestations and neuroimaging findings helps to validate our data. The neuroradiologists who reviewed MRI scans had gained extensive experience in COVID neuroimaging before scans were reviewed for this protocol, presumably enabling more legitimate differentiation between COVID-19-associated changes and clinically insignificant findings. Additionally, given that this was a retrospective study, we have missed important patient information including additional symptoms not documented in charts. It is possible that neurological manifestations were more prevalent than reported (e.g., anosmia) since patients were not prospectively screened for them.

Prospective studies will enhance our understanding of the neurological impact of COVID-19. Longitudinal studies that record patient status at multiple junctures will provide a better understanding of the disease’s effects on the brain. A recent MRI-based 3-month follow-up study used diffusion tensor imaging and 3D high-resolution T1-weighted sequences to identify persistent neurological symptoms and structural defects several months into recovery [44]. Another longitudinal study exploited 18F-FDG-PET/CT in 7 patients with COVID-19 encephalopathy at three different time points: in the acute phase, one month later, and six months later after recovery [45]. PET scans showed consistent hypometabolism in the orbitofrontal, dorsolateral, and mesiofrontal regions in a population with lingering cognitive and emotional disorders. In the future, imaging at higher resolutions such as 3 Tesla or even 7 Tesla would also be valuable, as would conducting volumetric MR analysis [46]. Including advanced sequences such as diffusion tensor imaging and resting state functional MRI would also enable visualization of additional brain networks and activity implicated in COVID-19. Improved understanding of brain imaging markers of COVID-19 may provide a method to assess the effect of therapeutic interventions that reduce thromboembolism and overall mortality in COVID-19, including steroids and aspirin [47, 48].

## Conclusion

The influence of COVID-19 on the brain has garnered increasing attention, with emerging evidence of its neurological complications. Recognition is critical for neurologists and radiologists alike in the diagnosis and treatment of COVID-19 patients. We report a diverse array of abnormalities that enhance understanding of the neurological ramifications of the disease and highlight several understudied patient populations, including cancer patients. In our cohort, the most common neurological manifestations included encephalopathy, impaired responsiveness/coma, and ischemic stroke, and the most frequent neuroimaging findings were non-ischemic parenchymal T2/FLAIR hyperintensities and acute/subacute infarcts.

No significant differences were observed in terms of neurological manifestations or neuroimaging findings in cancer versus non-cancer patients. Some neurological findings were significantly higher in the group with ventilatory support but are not similarly reflected in the pattern of neuroimaging abnormalities, but a larger sample size could help establish correlations between the increased neurological manifestations and neuroimaging findings in the group with ventilatory support. However, given the size of our cohort, further investigation with larger population is warranted to better elucidate the potential role of cancer in neurological manifestations due to COVID-19.

## Supporting information

S1 Dataset (XLSX)

## Abbreviations

ADC: apparent diffusion coefficient
CNS: central nervous system
COVID-19: coronavirus 2019
DWI: diffusion weighted imaging
MRI: magnetic resonance imaging
PCR: polymerase chain reaction
SARS-CoV-2: coronavirus severe acute respiratory syndrome 2
